# Characterization of Viscoelastic Performance and VOC Emission of Warm-Mixed SBS Asphalt Binder Under Different Dosages of Warm-Mixed Additive

**DOI:** 10.3390/ma19030485

**Published:** 2026-01-26

**Authors:** Wentao Wang, Yue Yang, Mengxue Xu, Xiangrui Han, Yinghao Miao, Linbing Wang

**Affiliations:** 1 National Center for Materials Service Safety, University of Science and Technology Beijing, Beijing 100083, China; wentaowang@ustb.edu.cn (W.W.);; 2 The Sensing and Perception Lab, School of Environmental, Civil, Agricultural and Mechanical Engineering, University of Georgia, Athens, GA 30602, USA

**Keywords:** warm-mixed asphalt binder, rotational viscosity, viscoelastic performance, VOC emission, additive dosage

## Abstract

Warm-mixed asphalt technology can significantly reduce the heating temperatures required for asphalt pavement construction, which makes it one of the crucial technical approaches in road engineering for achieving energy conservation and emission reduction, and carbon neutrality. Existing research often focuses on designing asphalt materials to ensure optimal service performance, but insufficient attention has been paid to the specific extent of reduction in asphalt fume emissions. However, the latter is a critical factor that cannot be neglected when constructing asphalt pavements in environmentally sensitive regions. Considering the environmental factor, this study systematically explores the comprehensive influence of different warm-mixed additive dosages on the viscoelastic properties and VOC emissions of warm-mixed SBS asphalt binder using rotational viscosity, bending beam rheometer (BBR), dynamic shear rheometer (DSR), and gas chromatography–mass spectrometry (GC-MS) test methods. The findings show that the application of warm-mixed additive does not compromise the comprehensive properties of SBS asphalt binder, but partially enhances its service performance instead. Due to the significant reduction in heating temperature, asphalt VOC emissions are indirectly reduced. Although the warm-mixed additive possesses a certain degree of volatility, its application still shows a significant trend toward emission reduction. Despite 0.4% being a relatively economical dosage of warm-mixed additive, a slight increase to 0.5% can achieve more pronounced environmental benefits in VOC emission reduction while maintaining comprehensive service performance that meets specification requirements. The findings can provide new insights for the application and decision-making of warm-mixed asphalt technology in environmentally sensitive regions.

## 1. Introduction

**Figure 1:**
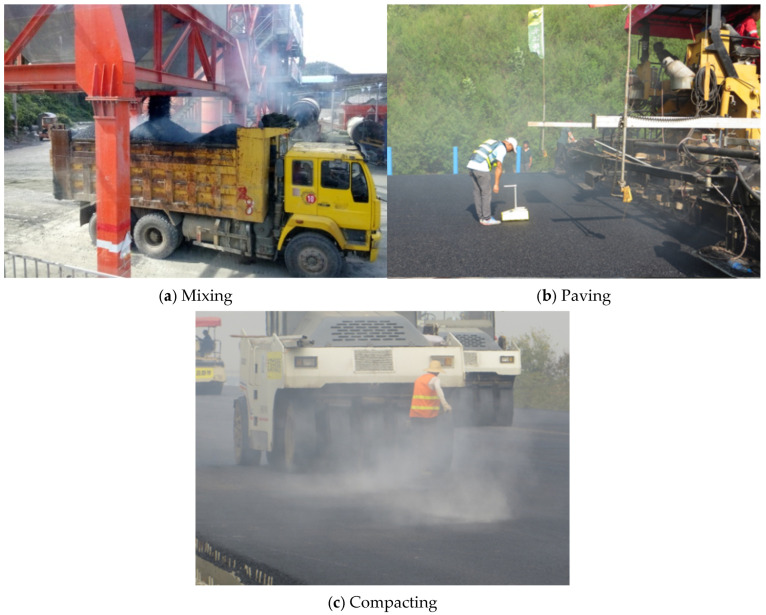
Obvious asphalt VOC emissions during construction procedures of asphalt layers.

Styrene–Butadiene–Styrene (SBS)-modified asphalt binder is commonly used in the middle and surface layers of asphalt pavements. During the construction process of asphalt pavement, raw materials such as asphalt and aggregate need to be heated and mixed at temperatures ranging from 150 °C to 180 °C, followed by processes of transportation, paving, and compaction at temperatures above 135 °C [1]. All these construction stages involve high-temperature heating, which leads to significant energy consumption and noticeable asphalt volatile organic compound (VOC) emissions [2,3], as shown in Figure 1. Warm-mixed asphalt technology serves as one of the crucial technical approaches in road engineering [4,5,6,7] for achieving energy conservation and emission reduction, as well as carbon neutrality [8,9]. It enables a substantial reduction in the required heating temperature for asphalt binder, thereby significantly decreasing energy consumption and carbon emissions during the construction process.

Warm-mixed asphalt technology is often applied to asphalt pavement, accompanied by environmentally friendly research topics. Li et al. [10] explored the effects of rubber absorption on the aging resistance of hot and warm asphalt rubber binders prepared with waste tire rubber. The warm-mixed additives were found to affect the asphalt rubber sample’s rheological properties, but its aging resistance was not compromised. Doyle et al. [11] compared hot- and warm-mixed asphalt rutting performance, and found that a hot asphalt mixture was similar to or slightly better than a warm-mixed asphalt mixture. Abd El-Hakim et al. [12] also discussed the difference between the moisture susceptibility of hot and warm mix asphalts in two aspects of laboratory and field experimental methods. The findings in their study indicated that small differences in moisture susceptibility were found between these two asphalt materials. Thus, the warm-mixed asphalt technology can satisfy the technical requirements of asphalt pavement materials well under the background of being green and low-carbon, and providing environmental protection.

Attention has also been paid to asphalt VOC emissions recently. Borinelli et al. [13] investigated the VOC emission features of commercial base asphalt and crumb-rubber-modified asphalt binder with a fixed dosage of crumb rubber particles. In their study, the influence of two heating processes (step-by-step raising and incremental increase) on VOC emission rates were evaluated. Lei et al. [14] discussed VOC emission at three temperatures with the rheological properties of different asphalt binder samples. They found that the complex-flow activation energy increased after VOC emission, while naphthalene content exhibited a better linear relation with activation energy. Mo et al. [15] found that hazardous potentials of asphalt fumes varied with asphalt source and mixing temperature. They suggested that proper material selection and construction strategies might be chosen for hazard mitigation.

Variable parameters concerning the VOC emissions of warm-mixed asphalt binder were often discussed, which mainly included categories of warm-mixed additive or base asphalt, warm-mixed additive dosages, heating duration, heating temperature values, stirring speed, and duration. However, these research studies did not explore the features of asphalt fume emissions from the perspective of engineering practice, but just discussed emission variation in variable parameters from an academic view. In the practical engineering application of warm-mixed asphalt technology, it is important to pay attention to how the service performance and emission characteristics of a warm-mixed asphalt mixture vary if the warm-mixed additive dosages are changed. Specifically, comprehensive effects of both heating temperature values and additive dosages should be taken into account to reach the same viscosity of warm-mixed asphalt binder for mixing, paving, and compacting processes, to ensure construction quality. Based on the analysis discussed above, research studies related to warm-mixed asphalt mixture primarily focused on designing asphalt materials to ensure optimal service performance, but insufficient attention has been paid to the specific extent of reduction in carbon-containing gas emissions, such as asphalt fumes. Yet, the latter is a crucial factor that must be considered for asphalt pavement construction in environmentally sensitive regions, such as urban residential zones, ecological reserves, and poorly ventilated (extra-long) tunnels [16,17,18]. As a result, warm-mixed asphalt technology often fails to deliver its full potential in terms of energy conservation, emission reduction, and environmental friendliness.

This study explored the comprehensive influence of different warm-mixed additive dosages on the viscoelastic properties and VOC emissions of warm-mixed SBS asphalt binder. Firstly, the viscosity–temperature characteristics of warm-mixed SBS asphalt binder were investigated using the rotational viscosity test method to establish experimental reference data for subsequent sample preparation. Subsequently, bending beam rheometer (BBR) and dynamic shear rheometer (DSR) test methods were employed to inspect the viscoelastic behaviors of asphalt materials at low and high temperatures, respectively. Finally, the gas chromatography–mass spectrometry (GC-MS) test method was applied to quantitatively analyze the composition of asphalt VOC emissions generated during the heating process, aiming to explore the emission characteristics associated with different warm-mixed additive dosages.

## 2. Materials and Sample Preparation

**Figure 2:**
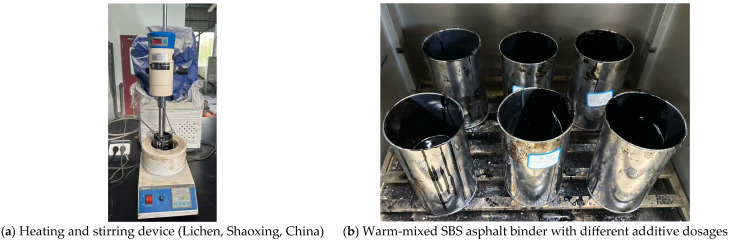
Fabrication of the warm-mixed SBS asphalt binder.

In this study, a typical SBS asphalt binder was selected to be the base material for warm-mixed modification with six additive dosages using a heating and stirring device, which include 0.0%, 0.2%, 0.3%, 0.4%, 0.5%, and 0.6% of the mass of the SBS asphalt binder, as shown in Figure 2. The warm-mixed additive applied in this study is a typical kind of surfactant admixture, which is developed by a local construction company in Beijing, China. Specifically, the 0.4% dosage is selected from the application of a warm-mixed asphalt mixture in an actual pavement constructing project. The basic physical properties of the SBS asphalt binder were inspected, and the relevant results are summarized in Table 1, which meet the requirements of the specification [19].

The base material was first heated in an oven at 150 °C for about 3 h and melted into liquid state, which was then moved and mounted into the heating device for further modification. To reach the same viscosity of the mixing process in an asphalt layer construction, warm-mixed SBS asphalt binder with different additive dosages should need corresponding different heating and stirring temperatures. Therefore, the rotational viscosity test method was first applied in this study to characterize the viscosity–temperature relationship, and then to determine the relevant temperature values to fabricate warm-mixed SBS asphalt binder with different additive dosages. Accurately weighed additives were poured into the base SBS asphalt binder and continuously stirred with a speed of 500 r/min for about 20 min. Then, the fabricated warm-mixed SBS asphalt binder was poured into specific molds to prepare samples for rotational viscosity, BBR, DSR, and GC-MS experiments.

## 3. Test and Analysis Methods

### 3.1. The Rotational Viscosity Test Method

**Figure 3:**
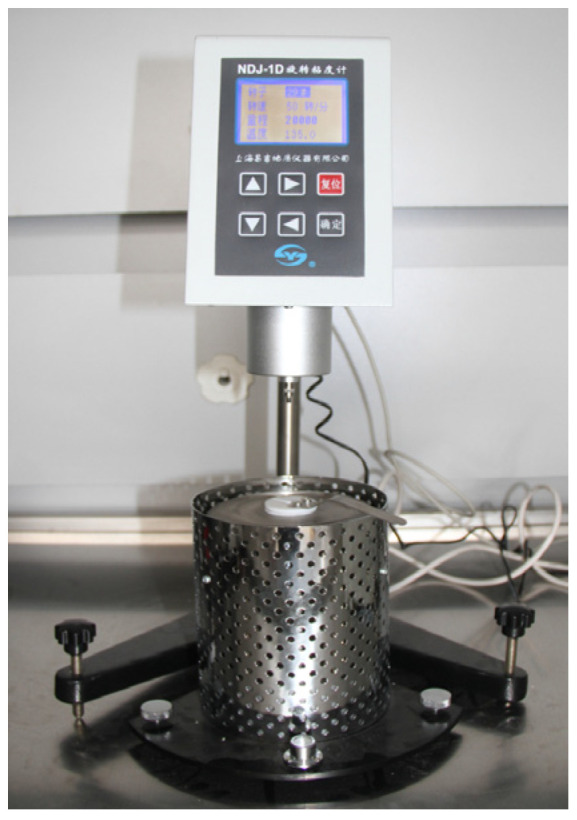
The rotational viscosity device (Changji, Shanghai, China).

Asphalt materials should be heated and melted into liquid state with suitable viscosity values for both mixing and compacting stages to ensure good construction quality. The rotational viscosity test method was applied to measure the viscosities of the warm-mixed SBS asphalt binder with different additive dosages at 135 °C and 175 °C, respectively, as shown in Figure 3, and then the viscosity–temperature curve could be determined [20,21]. For pavement petroleum asphalt materials, the viscosity ranges suitable for mixing and compacting procedures are 0.17 Pa·s ± 0.02 Pa·s and 0.28 Pa·s ± 0.03 Pa·s, respectively [19]. Two parallel samples of each warm-mixed additive dosage were prepared for rotational viscosity evaluation. In this study, the additive dosage of 0.4% was selected as the reference value due to its practical engineering application, and its viscosity value was thus referred to in order to determine the relevant heating temperatures for asphalt samples with different additive dosages.

### 3.2. The BBR Test Method

**Figure 4:**
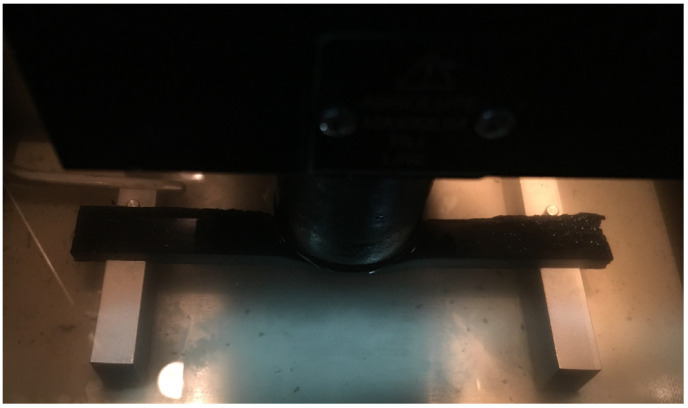
A sample of warm-mixed SBS asphalt binder in the BBR test.

The BBR method [22] was adopted to explore low-temperature viscoelastic properties of warm-mixed SBS asphalt binder with different additive dosages. As shown in Figure 4, a thin beam sample is located on two supports with a constant load of 980 mN ± 50 mN placed on its midspan, while the flexural deformation can be measured. The entire loading duration lasts about 240 s, while the BBR testing temperature was controlled at −12 °C in this study [23]. Three parallel samples of each warm-mixed additive dosage were prepared for BBR evaluation.

The basic indicators of creep stiffness *S* and creep rate *m-value* can be calculated based on testing data measured at the 60th second, which mainly include creep mechanical stress and strain. The creep stiffness *S* reflects the magnitude of stress generated in the asphalt binder under thermal influence. A relative lower value of creep stiffness *S* indicates stronger resistance to stress variations induced by temperature, which means a better cracking resistance. The creep rate *m-value* reflects the relaxation capacity of asphalt binder and is positively correlated with the stress release rate. A relative higher value of the creep rate *m-value* denotes greater relaxation capacity and superior cracking resistance. Creep stiffness *S* and creep rate *m-value* can be further used to derive the index of *m*/*S*, which reflects the growth rate of creep compliance *D*′(*t*), as shown in Equation (1). The detailed derivation processes of *m*/*S* can be found in the previous research [23,24]. A relative larger value of *m*/*S* often indicates a larger value of *D*′(*t*), which means a slower increase in creep stiffness *S*.

(1) $$\frac{m\left(t\right)}{S\left(t\right)}\approx D^{\prime }\left(t\right)\times t$$

The Burgers model, which contains characteristics of both the Maxwell and Kelvin models, can fit the creep compliance curve well in the BBR test. In the Burgers Equation (2) of creep compliance *D*(*t*), *E*_1_ and *E*_2_ are the instantaneous elastic modulus and delayed elastic modulus, respectively, while both *η*_1_ and *η*_2_ are viscous coefficients. These viscoelastic parameters can be further used to derive indexes of relaxation time *λ* and the delay time *τ*, which are calculated by Equations (3) and (4). Relaxation time λ reflects the asphalt sample’s ability to release internal stress, while delay time τ means creep and delayed elastic performance. A relative lower value of relaxation time *λ* indicates a better viscous performance, while a relative larger value of delay time *τ* usually means a much more viscous deformation, which means a better cracking resistance.

(2) $$D\left(t\right)=\frac{\varepsilon \left(t\right)}{\sigma_{o}}=\frac{1}{E_{1}}+\frac{t}{\eta_{1}}+\frac{1}{E_{2}}\left(1-e^{-t\cdot \frac{E_{2}}{\eta_{2}}}\right)$$

(3) $$\lambda =\frac{\eta_{1}}{E_{1}}$$

(4) $$\tau =\frac{\eta_{2}}{E_{2}}$$

### 3.3. The MSCR Test Method

**Figure 5:**
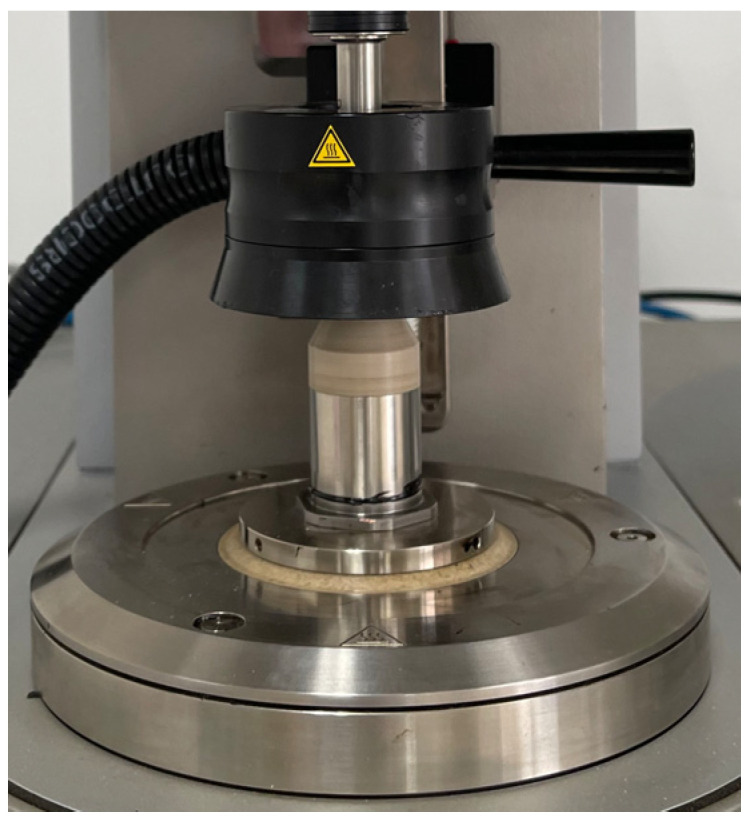
MSCR test using the DSR device (Anton Paar GmbH, Graz, Austria).

The DSR device was applied to conduct the multiple stress creep and recovery (MSCR) test [25,26] to evaluate the high-temperature rheological behaviors of warm-mixed SBS asphalt binder with different additive dosages, as shown in Figure 5. The testing temperature was controlled at 64 °C in this study. Two parallel samples of each warm-mixed additive dosage were prepared for MSCR evaluation. A MSCR test often contains 30 cycles, which include the first 10 cycles for the 0.1 kPa preloading period, the subsequent 10 cycles for the 0.1 kPa formal loading stage, and the remaining 10 cycles for the 3.2 kPa formal loading duration. Every cycle includes a loading period of 1 s followed by a recovering period of 9 s, and the entire MSCR test lasts for 300 s. The 0.1 kPa is applied to simulate a typical light traffic loading and obtain performance of the asphalt binder within the linear viscoelastic range. The value of 3.2 kPa is designated to simulate representative heavy traffic loading and detect the asphalt material’s nonlinear viscoelastic behaviors. The non-recoverable creep compliance *J_nr_* for every creep recovery cycle can be determined based on Equation (5), where *γ_nr_* and *γ*_0_ are the initial strain and residual strain, respectively, for each cycle, and *τ* is the shear stress. A relative lower value of non-recoverable creep compliance *J_nr_* means a stronger capacity of permanent deformation resistance. Meanwhile, a larger value of the creep recovery ratio *R* indicates a better ability of recovery resilience.

(5) $$J_{nr}=\frac{\gamma_{nr}-\gamma_{0}}{\tau }$$

### 3.4. The VOC Emission Test Method

**Figure 6:**
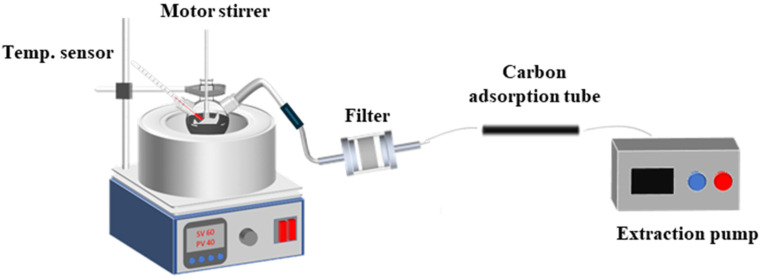
Schematic diagram of the asphalt VOC gas collection system.

Asphalt fumes generated during constructing processes often contain small particulate matters and VOCs, which not only negatively impact air quality at construction sites but also pose potential irritation and health risks to workers. Asphalt VOCs mainly include aliphatic hydrocarbons (alkanes and alkenes), aromatic hydrocarbons, and oxygenated hydrocarbon derivatives, as well as CO_2_, CO, NO_x_, and SO_2_. In this study, asphalt VOCs were generated and collected using the assembled test system, as shown in Figure 6.

**Figure 7:**
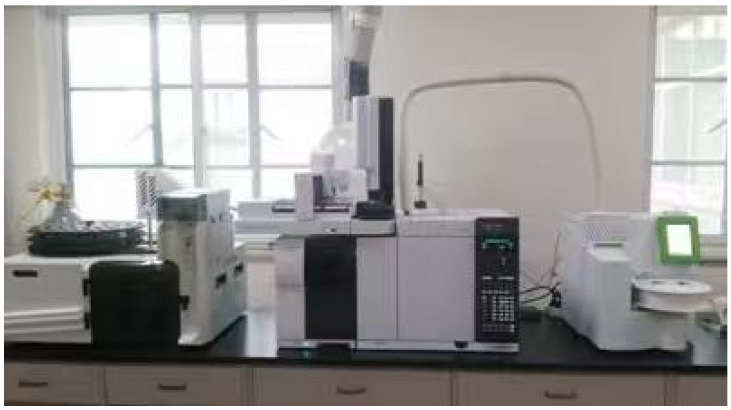
The GC-MS system (Agilent 7890B-5977B, Agilent Technologies Inc., Santa Clara, CA, USA).

For each group of warm-mixed SBS asphalt binder with different additive dosages, a 150 g sample was heated and maintained at its corresponding temperature for 30 min. The precision of temperature control is within 0.5 °C. To ensure a uniform heating effect, the asphalt sample was stirred at 1000 rpm throughout the process. An asphalt VOC sample of each warm-mixed additive dosage was prepared. The emitted asphalt fume was then drawn by an air pump at a flow rate of 0.5 L/min for 30 s and directed into an activated carbon adsorption tube for subsequent thermal desorption/purge-and-trap analysis using the GC-MS system, as shown in Figure 7. Specifically, small particulate matter present in the asphalt fumes was captured by the filter to prevent clogging the adsorption tube.

## 4. Results and Discussions

### 4.1. Viscosity–Temperature Characteristics

**Figure 8:**
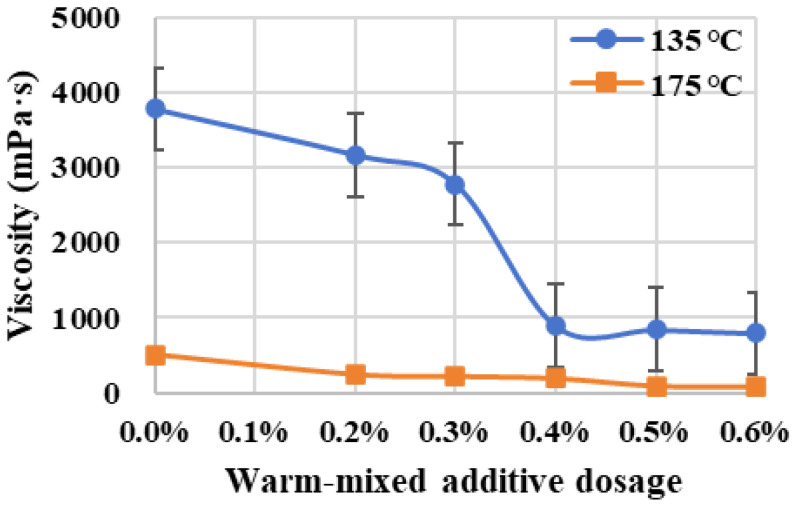
Viscosity variation in warm-mixed SBS asphalt binder with different additive dosages.

The viscosities of warm-mixed SBS asphalt binder with different additive dosages were determined at both 135 °C and 175 °C using the rotational viscosity test method, as shown in Figure 8. It is obviously proven that asphalt binder is a typical temperature-sensitive material. Compared with the control group of 0.0% warm-mixed additive dosage, the increase in dosages can significantly reduce the viscosities of SBS asphalt binder. At the relatively low temperature condition of 135 °C, the viscosities of warm-mixed asphalt binder show a clear decreasing trend as the additive dosages increase. In particular, when the dosage increases from 0.3% to 0.4%, a sharp and substantial drop trend in the viscosity curve is observed. At the relatively high temperature condition of 175 °C, although the control group’s viscosity is already at a relative lower level, a further noticeable improvement in viscosity reduction is still evident with the increasing warm-mixed additive dosages. Therefore, the warm-mixed additive used in this study has a significant effect on reducing the viscosity of the SBS asphalt binder, while 0.4% is a relatively economical dosage with a good viscosity reduction.

**Figure 9:**
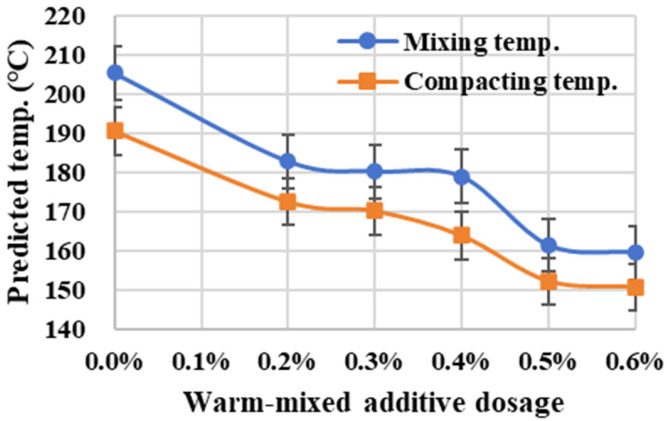
Predicted temp. variation in warm-mixed SBS asphalt binder with different additive dosages.

The viscosity–temperature curve is further applied to predict mixing and compacting temperatures according to the viscosity requirements of 0.17 Pa·s ± 0.02 Pa·s and 0.28 Pa·s ± 0.03 Pa·s, respectively [19], as shown in Figure 9. The predicted temperature curves exhibit decreasing trends with the increase in warm-mixed additive dosage. Specifically, the rate of temperature reduction slows within the dosage range of 0.2% to 0.4%. However, when the dosage is further increased from 0.4% to 0.5%, the mixing temperature still demonstrates a significant and substantial decrease. Therefore, the warm-mixed additive can effectively reduce the constructing temperatures of asphalt materials, and continually increasing the additive dosage from 0.4% to 0.5% yields a greater effect on temperature reduction.

The warm-mixed SBS asphalt binder with 0.4% additive dosage was already applied in a practical engineering project of asphalt pavement construction. In this case, its viscosity value is thus selected as a reference in this study to determine relevant mixing temperatures for samples with other additive dosages. The viscosity–temperature curve was employed to predict heating temperatures required for warm-mixed SBS asphalt binder at other additive dosages to achieve the same viscosity, which is 269.1 mPa·s, as shown in Table 2. Specially, the 0.0% dosage group’s temperature is predicted as 191.7 °C but exceeds the specification’s requirement of 175 °C [19], thus its mixing temperature was directly determined at 175 °C with the viscosity of 512 mPa·s. The temperature values summarized in Table 2 establish the testing foundation of sample fabrication for subsequent performance evaluation.

### 4.2. Low-Temperature Viscoelastic Performance

**Figure 10:**
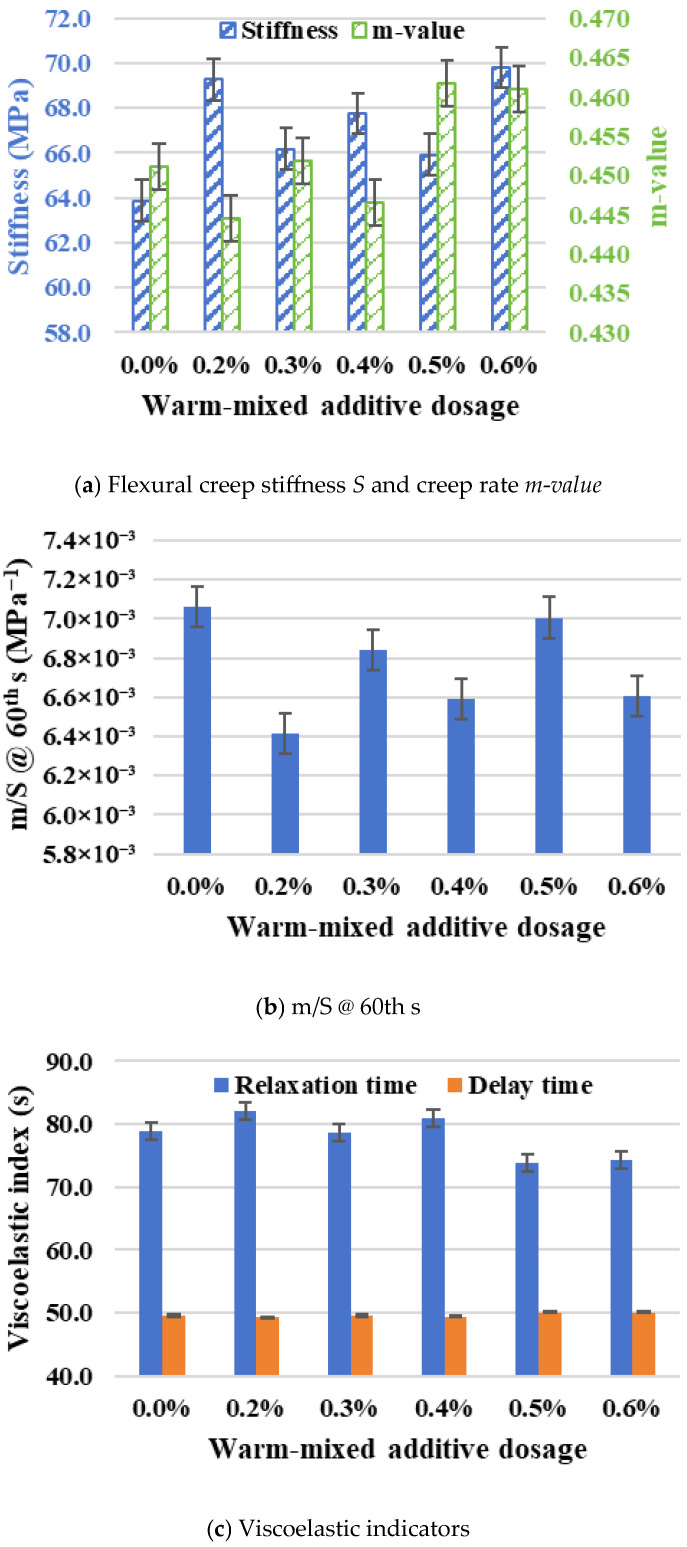
Variations in low-temperature indexes in BBR tests.

The variation in the low-temperature creep stiffness *S* and the creep rate *m-value* of warm-mixed SBS asphalt binder with different additive dosages can be observed in Figure 10a. The values of creep stiffness *S* for all groups are measured within the range of 60–70 MPa, which are significantly lower than the upper limit of 300 MPa according to the specification [22]. It indicates that the application of warm-mixed additive does not cause the failure of asphalt materials. Compared with the control group, the application of warm-mixed additive resulted in slight increases in creep stiffness *S*. The values of creep stiffness *S* rise sharply to 69.3 MPa at the 0.2% dosage, then remain essentially stable within 0.3–0.5%, and finally increase to 69.8 MPa. The values of creep rate *m-value* for all groups are above 0.44, exceeding the standard requirement of 0.30 [22]. This implies that the warm-mixed SBS asphalt binder possesses a good capacity for rapid thermal stress dissipation induced by sudden temperature drops. The values of the creep rate *m-value* fluctuate slightly in the range of 0.2–0.4% relative to the control group, but show a slight upward trend at dosages of 0.5–0.6%.

The indicator of *m/S @ 60th* is positively related to the growth rate of creep compliance, but shares an opposite fluctuation with creep stiffness *S*. As shown in Figure 10b, the variation in *m/S @ 60th* for groups of warm-mixed SBS asphalt binder with different additive dosages exhibit an exactly one-to-one opposite relationship with the creep stiffness *S*. This indicates that the warm-mixed additive makes asphalt binder slightly stiffer with an increasing trend of creep stiffness *S* or a decreasing trend of *m/S @ 60th*, but it also increases or maintains the creep rate *m-value*, thereby improving its ability to withstand thermal stress.

The viscoelastic indicators of relaxation time *λ* and time *τ* are further determined to evaluate low-temperature viscoelastic behaviors of warm-mixed SBS asphalt binder with different additive dosages. As shown in Figure 10c, the values of relaxation time *λ* basically show a downwards trend, which means the viscous performance of warm-mixed SBS asphalt binder increases gradually with the additive dosage. The values of delay time *τ* are slightly affected by the additive dosage. This indicates that higher additive dosages may help to add some viscous components of the asphalt binder, thereby further enhancing its cracking resistance.

Taking the testing data of 0.4% additive dosage as the reference level, values of creep stiffness *S* and creep rate *m-value* for other groups fluctuate within ±5%. Given the normal variability of experimental data in scientific research, warm-mixed additive dosage has no significant impact on the low-temperature creep performance of warm-mixed SBS asphalt binder in this study. The application of warm-mixed additive leads to a slight increase in low-temperature creep stiffness *S*, but the increase is limited and stabilizes with higher additive dosages. High dosages (>0.4%) further enhance the creep rate *m-value*, which improves the stress-relaxation capacity of asphalt binder. Taking the stability of low-temperature mechanical performance into account, the warm-mixed additive dosage range of 0.3–0.5% is strongly considered to be adopted.

### 4.3. High-Temperature Viscoelastic Performance

**Figure 11:**
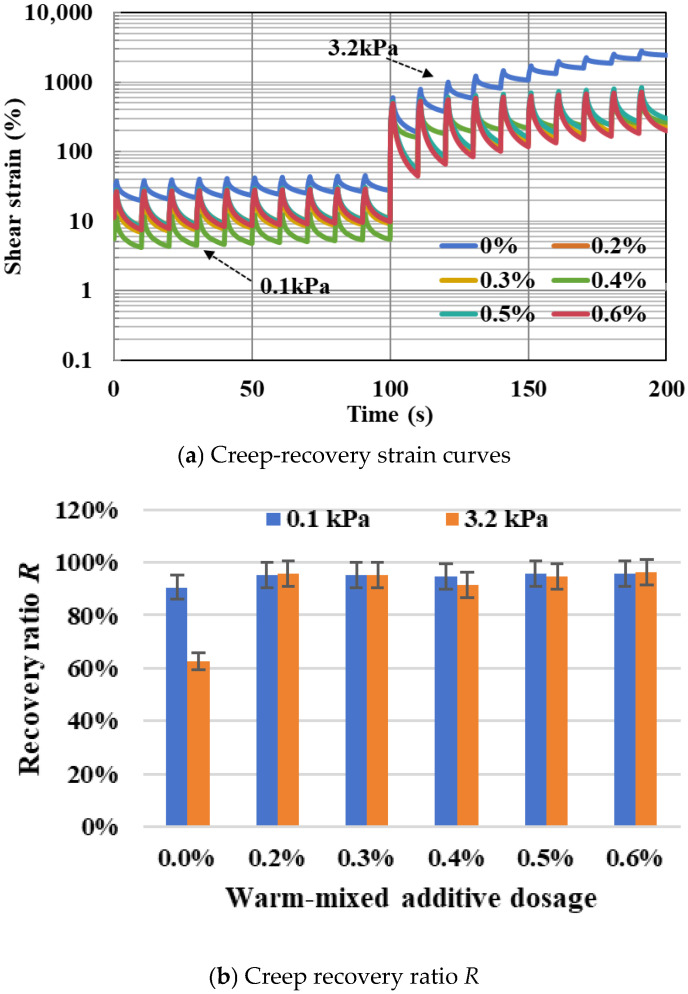

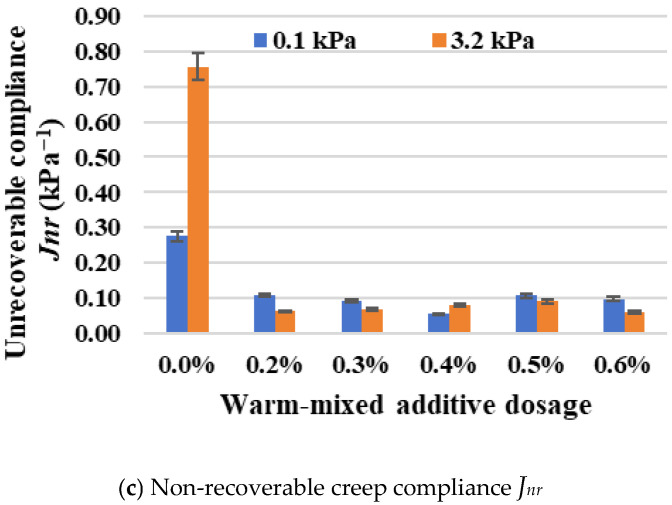
Variations in high-temperature indexes in MSCR tests.

Variations in the high-temperature viscoelastic performance of warm-mixed SBS asphalt binder are determined using the MSCR method and presented in Figure 11. As shown in Figure 11a, creep-recovery shear-strain curves of different warm-mixed additive dosages are closely clustered in the 0.1 kPa loading period and visually illustrate no significant differences. This indicates that within the linear viscoelastic range under a relative light traffic loading, additive dosage has a minor effect on the high-temperature rheological properties of the warm-mixed SBS asphalt binder. However, the deformation resistance of different asphalt samples exhibits a notable difference when the MSCR test enters the 3.2 kPa heavy traffic loading stage. The control group shows a rapid growth in cumulative shear deformation with its curve positioned higher and limited recovery after unloading, which indicates a relatively weak high-temperature shear deformation resistance. In contrast, warm-mixed groups are tightly clustered near the bottom of the Figure, which exhibits relatively small peak values of shear strain and significant elastic recovery characteristics.

The variations in non-recoverable creep compliance *J_nr_* and creep recovery ratio *R* are shown in Figure 11b,c. In the 3.2 kPa heavy traffic loading stage, the control group without warm-mixed modification exhibits a high *J_nr_* value of 0.76 kPa^−1^ and a low *R* value of 62.46%, but all modified groups show significant reductions in *J_nr_* values to below 0.1 kPa^−1^ and obvious increases in *R* to above 91%. The *J_nr_* values show a first increasing and then decreasing trend, and the *R* values exhibit the opposite trend to the additive dosage, while both the testing values of *J_nr_* and *R* for all warm-mixed groups do not differ much. Specifically, both warm-mixed SBS asphalt binder samples with additive dosages of 0.4% and 0.5% show better relative high-temperature behaviors. The application of warm-mixed additive significantly enhances the high-temperature deformation resistance of SBS asphalt binder, while the improving effect is not obviously sensitive to the warm-mixed additive dosage.

### 4.4. VOC Emission Characteristics

**Figure 12:**
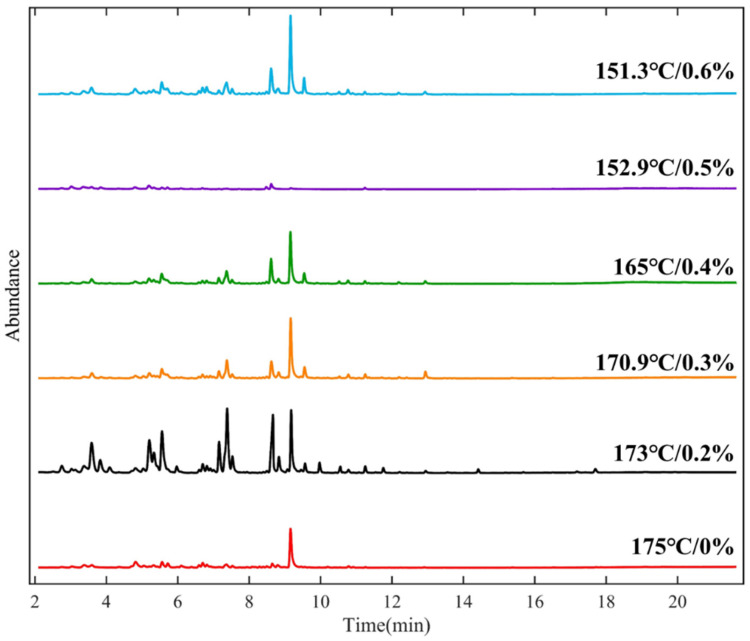
GC-MS chromatograms of warm-mixed SBS asphalt binder at different temperatures.

The application of warm-mixed additive in SBS asphalt binder can decrease the heating temperature required to reach a certain viscosity. Quantitative analysis on asphalt VOCs using the GC-MS method can systematically reveal the coupling influence mechanism of temperature and warm-mixed additive dosage on asphalt VOC emissions. As shown in Figure 12, the GC-MS chromatograms indicate that the retention time ranges of chromatographic peaks generally remain stable under different temperature conditions. This means that the main volatile components possess relatively fixed molecular structures and diffusion characteristics. However, both chromatographic peaks’ numbers and intensities rise significantly as the temperature increases, which means an increase in the variety of volatiles and a continuous enhancement in emission levels. The experimental analysis identifies nearly one hundred organic compounds from asphalt VOC emissions. These chemical compositions become more complex as heating temperatures increase. This is not only related to the accelerated diffusion rate of light fractions in asphalt VOCs but also potentially linked to partial thermal decomposition of both the SBS modifier and the warm-mixed additive at elevated temperatures. The warm-mixed additive itself contains a certain content of oxygenated components that are prone to volatilization upon the heating process. Its application increases the variety of volatile components at a relative lower temperature, such as 150 °C. When the warm-mixed additive dosage is reduced, a raised heating temperature is required for asphalt binder to achieve the same mixing viscosity. It often leads to a substantial release of hydrocarbon and aromatic components. This indicates a clear indirect coupling effect between warm-mixed additive dosage and heating temperature.

**Figure 13:**
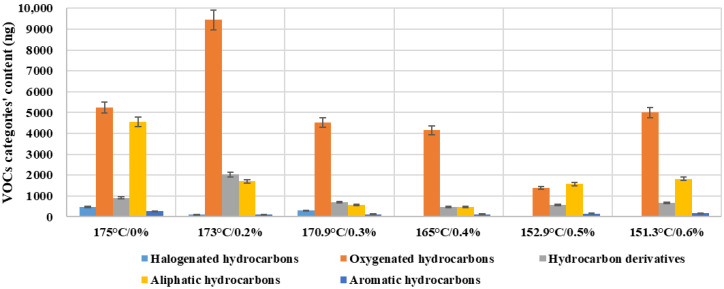
VOC categories’ contents of warm-mixed SBS asphalt binder with different additive dosages.

The proportion variation in different categories of VOCs exhibits significant selective release characteristics as temperature increases, as shown in Figure 13. This reflects the combined influence of activation energy differences and chemical structural variations on the volatilization behavior of each emission component. At relatively low temperatures, oxygenated hydrocarbons account for a notably higher proportion (70–80%). This can be attributed to their higher vapor pressure, weaker binding capacity, and contributions from the thermal decomposition products of warm-mixed additives. Simultaneously, the volatilization rates of hydrocarbons and aromatic hydrocarbons decline more rapidly at low temperatures, which leads to the proportional dominance of oxygenated hydrocarbons. As temperature increases, the release of aliphatic and aromatic hydrocarbons becomes more intense. This is primarily due to the promotion of the thermal cracking of light hydrocarbon fractions, the desorption and migration of aromatic ring structures under high-temperature conditions, and the reduction in diffusion resistance resulting from the decreased viscosity of asphalt [27,28,29,30]. The rapid increase in the proportion of aromatic hydrocarbons in the high-temperature range carries greater environmental and health risk implications. It indicates that elevated temperatures not only increase the total emission volume but also raise the relative proportion of highly toxic components. Under identical sampling conditions, the total VOC emissions show a clear mitigation trend relative to the hot-mix control (175 °C/0%). Specifically, total VOCs decrease by 45.9% at 170.9 °C (0.3%), 54.6% at 165 °C (0.4%), and 67.8% at 152.9 °C (0.5%). In contrast, 173 °C causes a 16.3% increase, whereas 151.3 °C achieves only a 33.5% reduction.

**Figure 14:**
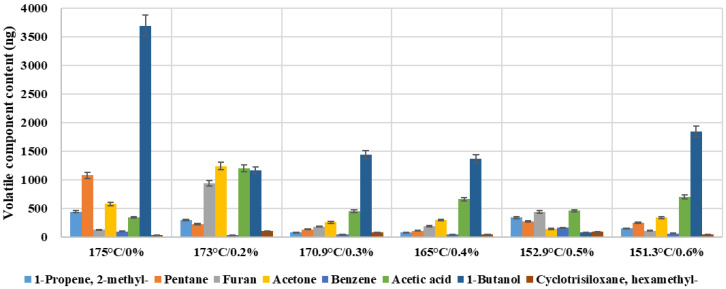
Typical volatile component contents of warm-mixed SBS asphalt binder with different additive dosages.

Figure 14 exhibits content variation in several typical volatile components of warm-mixed SBS asphalt binder with different additive dosages. The total amount of VOC emission increases as temperature rises, while a concentrated emission peak was observed around 173 °C, which may be formed from the superposition of multiple mechanisms. Around the temperature range of 170 °C–180 °C, initial thermal decomposition may occur in the SBS modifier, partial surfactant-type components in the warm-mixed additive reach their volatilization peak, the vapor pressure of C_6_–C_12_ hydrocarbons in the asphalt binder rises to a relative high level, and a significant drop in the asphalt binder’s viscosity may lead to a sharp reduction in the internal diffusion resistance. These factors work together to make asphalt VOC emissions sensitive to a temperature around 173 °C, and prolonged exposure of asphalt to this temperature during construction should be avoided. Some individual compounds still exhibit relatively high concentrations at lower temperatures, such as 1-Butanol. This indicates that VOC emissions are not only a thermally controlled equilibrium process but may also involve secondary pathways, such as the structural breakdown of the warm-mixed additive and oxidative side-reactions of the asphalt binder. It reflects the multi-source, composite release characteristics of asphalt VOC emissions in this study.

A data-driven assessment supports 0.3–0.5% as the optimal dosage. Compared with the control group of 0.0% dosage, the total emissions of the experimental group decreased by 45.9% (170.9 °C/0.3%), 54.6% (165 °C/0.4%), and 67.8% (152.9 °C/0.5%), respectively. On the contrary, the 173 °C/0.2% condition increased by 16.3% compared with the control group, indicating that 173 °C is an emission-sensitive temperature. Furthermore, increasing the dosage to 0.6% does not lead to further mitigation; only a 33.5% reduction in total VOCs, and oxygenated VOCs remained at a high level. This means additional volatilization or decomposition products generated from the additive itself. Therefore, 0.3–0.5% provides substantial mitigation via temperature lowering while avoiding additive-driven emission rebound.

From an environmental and health perspective, the identified VOC spectrum covers light alkanes/alkenes (e.g., pentane and methylpropene), oxygenated VOCs (e.g., acetone, acetic acid, and 1-butanol), heteroaromatics (e.g., furan), and aromatics (e.g., benzene) (Figure 14). These species are not equivalent in risk: aromatics and heteroaromatics are typically associated with stricter occupational exposure limits and higher chronic health concern, whereas oxygenated VOCs often contribute to acute irritation and odor nuisance. In addition, both aliphatic and oxygenated VOCs are key precursors for photochemical ozone formation and secondary organic aerosol, implying that emission mitigation benefits extend beyond the work zone to the surrounding air quality.

Although the laboratory collection configuration is not intended to replicate onsite dilution or ventilation, the fixed sampling volume enables an upper-bound estimation of source-stream concentration. For priority toxicants, benzene remains at the sub-mg·m^−3^ level, while furan shows a stronger temperature and additive sensitivity, highlighting that risk management should consider toxicity-weighted species rather than only the total mass. Therefore, it is suggested to control dwell time within the emission-sensitive temperature range (170–180 °C), and to ensure local exhaust ventilation (especially in enclosed environments, such as tunnels). It is also recommended to facilitate compliance with workplace air-quality requirements and to support safer infrastructure construction.

Overall, temperature is the dominant factor influencing asphalt VOC emissions. Reducing heating temperature can decrease the total VOC emissions by 60–80% and significantly lower the proportion of high-risk components such as aromatic hydrocarbons. It thereby achieves dual mitigation of both emission volume and hazards. Warm-mixed additive indirectly reduces VOC emissions by lowering the heating temperature. Although the warm-mixed additive used in this study possesses a certain degree of volatility, its application still shows a significant trend toward emission reduction. Considering factors of emission volume, chemical composition, and construction performance, the warm-mixed additive dosage range of 0.3–0.5% demonstrates optimal performance. It not only significantly reduces the heating temperature but also avoids excessive volatile by-products from the warm-mixed additive itself. This study systematically validates the effectiveness of warm-mixed technology in suppressing asphalt VOC emissions during asphalt layer construction. By elucidating thermal action mechanisms and behavioral differences in various volatile components, it provides a more theoretically grounded scientific basis for green road construction.

## 5. Conclusions

This study explored the comprehensive influence of different warm-mixed additive dosages on the viscoelastic properties and VOC emissions of a warm-mixed SBS asphalt binder. Based on the analysis discussed above, the following conclusions can be obtained:

(1) The warm-mixed additive could effectively reduce viscosity and construction temperature of the SBS asphalt binder. A dosage of 0.4% was relatively economical with a good viscosity reduction, but continually increasing additive dosage from 0.4% to 0.5% yielded a greater effect on temperature reduction.
(2) Increasing trends of creep stiffness *S* and creep rate *m-value* were determined within ±5% fluctuation, which indicated no significant impact of additive dosage on low-temperature creep performance. Higher additive dosages might help to add some viscous components of the asphalt binder, thereby further enhancing its cracking resistance.
(3) All warm-mixed groups showed significant reductions in *J_nr_* values to below 0.1 kPa^−1^ and obvious increases in *R* to above 91%. The improvement effect of high-temperature performance was not obviously sensitive to additive dosage. Dosage ranges from 0.4% to 0.5% showed better relative high-temperature behaviors.
(4) Temperature is the dominant factor influencing asphalt VOC emissions. Considering the factors of emission volume, chemical composition, and performance, the additive dosage range of 0.3–0.5% demonstrated optimal performance by reducing heating temperature and avoiding excessive volatile by-products from the additive itself.
(5) Despite 0.4% being a relatively economical dosage of warm-mixed additive, a slight increase to 0.5% could achieve more pronounced environmental benefits in VOC emission reduction while maintaining comprehensive service performance that met specification requirements.
(6) Asphalt VOC emissions are also affected by the aggregate gradation and the interaction between mixture and construction machinery, while these influential factors should be taken into account in future studies.

## Figures and Tables

**Table 1:** Physical properties of the SBS asphalt binder sample.

Items		Units	Requirements	Results
Penetration (25 °C, 100 g, 5 s)		0.1 mm	60–80	69
Softening point		°C	≥75	78.0
Ductility (5 cm/min)		cm	≥30 (5 °C)	39
Residue after rolling thin film oven test (163 °C, 85 min)	Mass loss	%	≤±1.0	−0.221
	Residual penetration ratio (25 °C, 100 g, 5 s)	%	≥60	68.1
	Residual ductility (5 cm/min)	cm	≥20 (5 °C)	23

**Table 2:** Suitable heating temperature values for warm-mixed SBS asphalt binder with different additive dosages to research the same viscosity (except for 0.0% dosage).

Warm-Mixed Additive Dosage	Viscosity	Heating and Stirring Temperature
0.0%	512 mPa·s	175.0 °C
0.2%	269.1 mPa·s	173.3 °C
0.3%		170.9 °C
0.4%		165.0 °C
0.5%		152.9 °C
0.6%		151.3 °C

## Data Availability

The original contributions presented in this study are included in the article. Further inquiries can be directed to the corresponding authors.
